# MMP‐13 binds to platelet receptors αIIbβ3 and GPVI and impairs aggregation and thrombus formation

**DOI:** 10.1002/rth2.12088

**Published:** 2018-03-25

**Authors:** Joanna‐Marie Howes, Nicholas Pugh, Samir W. Hamaia, Stephanie M. Jung, Vera Knäuper, Jean‐Daniel Malcor, Richard W. Farndale

**Affiliations:** ^1^ Department of Biochemistry University of Cambridge Cambridge UK; ^2^ Department of Biomedical and Forensic Sciences Anglia Ruskin University Cambridge UK; ^3^ Cardiff University Dental School Cardiff UK

**Keywords:** GPVI collagen receptor, Integrin alphaIIbbeta3 (αIIbβ3), matrix metalloproteinase‐13, platelets, thrombosis

## Abstract

**Essentials**

- MMP‐13 has the potential to influence platelet function and thrombus formation directly.
- We sought to elucidate whether MMP‐13 is able to bind to specific platelet receptors.
- MMP‐13 is able to bind to platelet alphaIIbbeta3 (αIIbβ3) and glycoprotein (GP)VI.
- These interactions are sufficient to inhibit platelet aggregation and thrombus formation.

**Background:**

Acute thrombotic syndromes lead to atherosclerotic plaque rupture with subsequent thrombus formation, myocardial infarction and stroke. Following rupture, flowing blood is exposed to plaque components, including collagen, which triggers platelet activation and aggregation. However, plaque rupture releases other components into the surrounding vessel which have the potential to influence platelet function and thrombus formation.

**Objectives:**

Here we sought to elucidate whether matrix metalloproteinase‐13 (MMP‐13), a collagenolytic metalloproteinase up‐regulated in atherothrombotic and inflammatory conditions, affects platelet aggregation and thrombus formation.

**Results:**

We demonstrate that MMP‐13 is able to bind to platelet receptors alphaIIbbeta3 (αIIbβ3) and platelet glycoprotein (GP)VI. The interactions between MMP‐13, GPVI and αIIbβ3 are sufficient to significantly inhibit washed platelet aggregation and decrease thrombus formation on fibrillar collagen.

**Conclusions:**

Our data demonstrate a role for MMP‐13 in the inhibition of both platelet aggregation and thrombus formation in whole flowing blood, and may provide new avenues of research into the mechanisms underlying the subtle role of MMP‐13 in atherothrombotic pathologies.

## 1 INTRODUCTION

Platelet‐extracellular matrix and platelet–platelet adhesions are central to the formation of thrombi. MMP‐13 is up‐regulated in inflammation, and is elevated in the atherosclerotic plaque, contributing to its vulnerability.^1^ It is also implicated in the progression and remodelling of cerebral tissue in stroke.^2^ Plaque rupture releases MMP‐13 into the local environment where it has direct access to plasma proteins, blood cells, and platelets. Following injury to the blood vessel wall, specific platelet receptors mediate platelet–collagen and platelet–platelet interactions. GPIbα binds to immobilized von Willebrand factor (VWF) in the vessel wall, initiating platelet capture,^3^ and glycoprotein (GP)VI binds directly to collagen and activates platelets. Integrin α2β1 stabilises the early stages of the platelet‐collagen interaction, and integrin αIIbβ3 supports platelet–platelet interactions mediated by fibrinogen and VWF.^4^, ^5^, ^6^, ^7^

MMP‐2 and ‐9 have previously been shown to bind to platelet receptors and/or to modulate platelet function.^8^, ^9^, ^10^, ^11^, ^12^, ^13^ Here, we hypothesized that MMP‐13 may also interact directly with platelet receptors GPVI, integrin α2β1, or the platelet adhesive integrin αIIbβ3 to modulate platelet adhesion, aggregation and thrombus formation. Our work identifies potential roles for MMP‐13 in modulating the recruitment or activation of platelets in thrombotic pathologies.

## 2 METHODS

### 2.1 MMP‐13 expression, purification and activation

ProMMP‐13 and its MMP‐13 catalytic (CAT, 249‐451) domain were expressed, purified, activated, and dialysed as previously described.^14^, ^15^, ^16^ The structurally homologous but catalytically inactive proMMP‐13(E204A) was a kind gift from Dr. R. Visse (Kennedy Institute of Rheumatology Division, Imperial College London, London, UK). GST‐Hemopexin (HPX) domain (264‐471) was expressed in *E. coli* using the pGEX‐2T expression vector, the forward primer TCCGCGTGGATCCCTCTATGGTCCAGGAGATGAA and the reverse primer GCAA‐ATTCCATTTTGTGGTGTTGAAGAATTCAT, which contain BamHI and EcoRI restriction sites respectively, as previously described.^16^

### 2.2 Washed platelet preparation and platelet adhesion assays

Plates were coated with 10 μg/ml MMP‐13 variants in Tris buffered saline (TBS) for 1 h at 24°C. Plates were then blocked with 5% BSA in TBS for 20 minutes at 24°C and washed with TBS prior to the addition of washed platelets. Platelets were purified and adhesion assays conducted as previously described.^17^, ^18^ Glanzmann thrombasthenic blood was kindly provided by Prof M. Makris, Royal Hallamshire Hospital, Sheffield, UK. GR144053 (4‐[4‐[4‐(aminoiminomethyl)phenyl]‐1‐piperazinyl]‐1‐piperidineacetic acid hydrochloride trihydrate) was purchased from Calbiochem, Nottingham, UK. The α2β1 integrin‐binding peptide GFOGER (GPC[GPP]5‐GFOGER‐[GPP]5‐GPC) and GPVI‐binding peptide CRP‐XL (GCO‐[GPO]10‐GCOG); cross‐linked where appropriate and the inert GPP10 (GPC‐[GPP]10‐GPC) were generated as previously described^7^ along with the anti–GPVI scFvs 10B12 and 1C3 and the non‐GPVI‐binding scFv 2D4^19^, ^20^, ^21^, ^22^, ^23^ which were a kind gift from Dr. P. Smethurst. Human fibrinogen type I was purchased from Sigma, UK. Anti–α2β1 antibody 6F1 was a kind gift from Prof. B. Coller (Mount Sinai Hospital, New York, NY, USA). RGDS (Arg‐Gly‐Asp‐Ser) and cyclic RGD (H‐Cys‐Arg‐Gly‐Asp‐Phe‐Pro‐Ala‐Ser‐Ser‐Cys‐OH) were purchased from Bachem, Weil am Rhein, Germany. The fibrinogen‐derived peptide, Lys‐Gln‐Ala‐Gly‐Asp‐Val (KQAGDV), was purchased from Innovagen, Sweden. Inhibitory antibodies/compounds were used at 10 μM (6F1, 10B12, 1C3, 2D4, cRGD, GR144053, KQAGDV) or 100 μmol L^−1^ (fibrinogen and RGDS).

### 2.3 Flow cytometry

In activation experiments, whole blood diluted 1:4 with Hepes buffered saline (HBS) was mixed for 10 minutes at 24°C with an equal volume of 10 μg/ml mouse anti P‐Selectin (Abcam, Cambs, UK) and the following agonists 2 mmol L^−1^ proMMP‐13(E204A) or MMP‐13, 100 μg/ml CRP‐XL, 100 μg/ml HORM^®^ equine collagen I fibers (Takeda, Linz, Austria), thrombin activating peptide (TRAP; 500 μmol L^−1^; Sigma, UK) calcium ionophore A23187 (100 μmol L^−1^; Sigma UK) or HBS (negative control) added. Alexa 488 conjugated anti‐mouse (30 μg/ml final concentration; Jackson Immuno Research, Ely, UK) was then added and after 10 minutes at 24°C the volume was made up to 500 μl with isotonic solution. After 30 minutes fluorescence was measured using an Accuri C6 flow cytometer (BD Biosciences, Oxford, UK). In inhibition experiments, whole blood was pre‐incubated with proMMP‐13(E204A), GR144053 (20 μmol L^−1^) or 10B12 (10 μg/ml) for 20 minutes prior to the addition of CRP‐XL.

### 2.4 Solid phase adhesion assays

Recombinant human αIIbβ3 and GPVI monomer were obtained from R&D Systems (Abingdon, Oxford, UK). Recombinant extracellular domain of GPVI (GPVIex, comprising D1D2 (amino acids 1–214; 42 kDa) fused with the Fc domain of human IgG (GPVI‐Fc2, 150 kDa) was prepared as previously described.^24^

HB 96‐well plates (Nunc, Langenselbold, Germany) were coated with recombinant GPVI monomer or dimer (10 μg/ml in Phosphate‐Buffered Saline [PBS]) for 1 h at 24°C. All further incubations were performed at room temperature for 1 h unless otherwise stated. The wells were washed three times with adhesion buffer (1 mg/ml BSA in PBS containing 0.1% [v/v] Tween‐20) between each incubation step. The wells were then blocked with 50 mg/ml BSA in TBS prior to the addition of MMP‐13 at a concentration of 83 nmol L^−1^ (unless otherwise stated) for 1 h at 24°C in adhesion buffer. Rabbit anti‐MMP‐13, raised against MMP‐13 hinge region (Abcam, Cambridge, UK), and goat anti‐rabbit HRP (Dako, Stockport, UK) were added at a dilution of 1:2000 in adhesion buffer prior to the addition of a TMB substrate system (Sigma, UK) and the plates read at 450 nm.

### 2.5 Aggregometry

Washed platelet aggregation was performed using a Chrono‐Log turbidimetric aggregometer (Labmedics, Abingdon on Thames, UK). 250 μL aliquots of platelets, 2 × 10^8^/mL in calcium‐free Tyrodes buffer (CFT), were pre‐incubated for 1 h with 80 nmol L^−1^ MMP‐13 or vehicle control prior to the addition of receptor agonists in a maximum volume of 5 μL. Thrombin, calcium ionophore A23187 (San Diego, CA, USA), bovine collagen I fibers (Ethicon Corp, Somerville, NJ, USA), HORM^®^ and CRP–XL were prepared and employed to activate platelets as previously described.^25^, ^26^ Aggregations were allowed to proceed for 5 minutes.

### 2.6 Cleavage of platelet receptors and their substrates by MMP‐13

Recombinant human (rh)GPVI, purified αIIbβ3 (100 μg/mL, R&D Systems) and human fibrinogen type I (1 mg/mL) were incubated with MMP‐13 or MMP‐13(E204A) (8 μmol L^−1^ final concentration) for 2 h at 37°C. An equal volume of Tris buffer was used as a negative control. Reducing sample buffer was then added to the mixture in preparation for electrophoresis and Western blotting.

### 2.7 In vitro sheddase activity assays

Dialysed MMP‐13 at a final concentration of 130 nmol L^–1^ was incubated with washed platelets for 60 minutes at 37°C. Positive controls for shedding included thrombin (1 U/mL, Sigma, UK) combined with fibrous type I collagen (1 mg/mL), the calcium ionophore A23187 (1 μg/mL). The platelets were then pelleted at 1500 g for 1 minute. The supernatants were aspirated and centrifuged again to ensure platelet depletion. This new supernatant was retained for analysis. Where indicated, platelet lysate was resuspended in reducing sample buffer.

### 2.8 Electrophoresis and Western blotting

Protein samples in reducing sample buffer were boiled for 5 minutes and applied to 4‐12% NuPage Gels and separated by electrophoresis using the Xcell SureLock system (Invitrogen, Paisley, UK) under reducing conditions. Proteins were then transferred on to nitrocellulose membrane (Millipore, Bedford, UK) at 40 V overnight at 4°C using a Mini Protean II system (Bio‐Rad, Hemel Hempstead, UK). Following transfer, the PVDF was blocked (5% nonfat dry powdered milk, 0.1% Tween 20 in TBS) for 1 h and primary antibody was then added (1:1000 dilution) and incubated for 2 h at room temperature. Anti‐human GPVI was a kind gift from Dr. P. Smethurst, and anti‐β3 was obtained from Abcam, Cambridge, UK. Following washes with TBST, the membrane was incubated with HRP conjugated secondary antibody (1:10000 dilution/TBST) for 1 h at 24°C. The PVDF was developed using a chemiluminescent substrate (GE Healthcare, Amersham, Bucks, UK).

### 2.9 Whole blood perfusion experiments

Whole blood was pre‐incubated with either carrier (TBS) or 80 nmol L^−1^ MMP‐13 for 1 h prior to perfusion over 10 μg/mL type I fibrous collagen as previously described.^17^, ^25^ Where indicated, slides were coated with MMP‐13(E204A) alone as a (negative) control.

## 3 RESULTS

**Figure 1:**
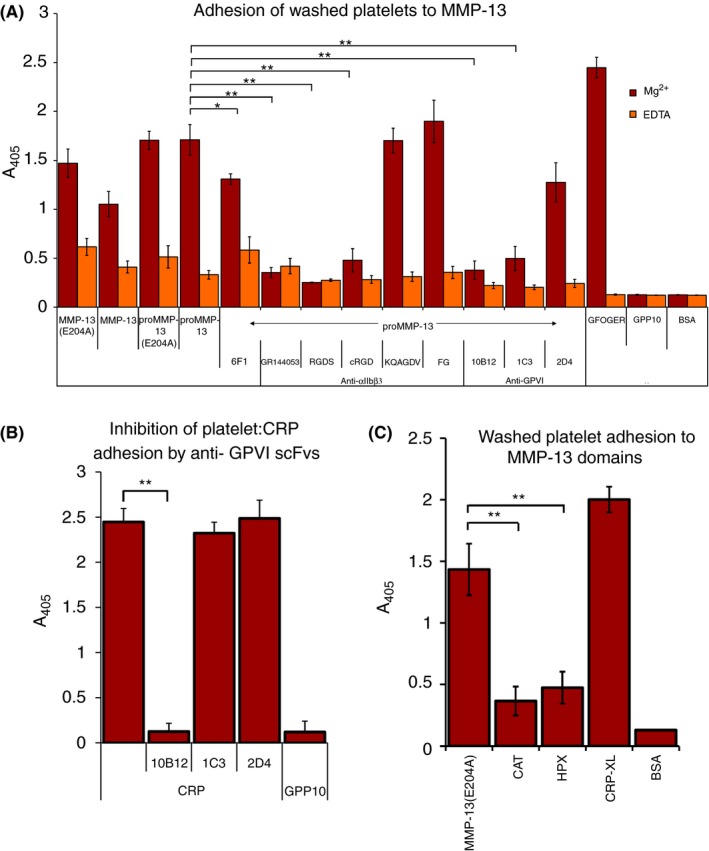
Washed platelet adhesion assays. (A) Platelets adherent to 10 μg/ml coated MMP‐13 variants in the presence of 2 mmol L^−1^ Mg^2+^ (red bars) or 2 mmol L^−1^ EDTA (orange bars) where stated. Where appropriate, platelets were pre‐incubated with anti‐GPVI, αIIbβ3 or α2β1 antagonists. BSA and GPP_10_ were used as Mg^2+^‐independent negative controls. The platelet α2β1 binding‐peptide GFOGER was included as an Mg^2+^ dependent positive control. *P < .05; **P < .01; ^†^(one‐way anova and Holm multiple comparison test) relative to untreated platelets in either the presence of Mg^2+^ or EDTA, as appropriate. (B) Inhibition of platelet adhesion to CRP by anti‐GPVI scFvs as described above (C) Platelets adherent to MMP‐13(E204A) and MMP‐13 CAT and HPX domains. CRP‐XL was used as a positive control. **P < .01 (one‐way anova and Holm multiple comparison test) relative to adhesion to MMP‐13(E204A). Data represent mean A_405_ ± SE of three experiments

Adhesion assays were performed in the presence of 2 mmol L^−1^ EDTA or Mg^2+^ to ablate or support integrin‐mediated adhesion. Platelet adhesion to MMP‐13 preparations was significantly reduced, but not abolished, by EDTA, suggesting both integrin‐dependent and ‐independent contributions, whereas EDTA fully abolished binding to the collagen‐binding integrin‐specific peptide GFOGER (Figure 1A).

Platelet pre‐incubation with the αIIbβ3 antagonists, GR144053, cRGD, and RGDS, and with anti‐GPVI scFv 10B12 and 1C3, all caused a substantial and significant reduction (*P *<* *.01) in platelet adhesion to proMMP‐13 (Figure 1A), with residual adhesion being observed in the presence of EDTA remaining above negative control levels (nonspecific substrates). This may indicate cooperative binding to αIIbβ3 and GPVI. Interaction between MMP‐13 and integrin α2β1 was less prominent, since blocking antibody 6F1 had just a small effect, and was not studied further. Platelet pre‐incubation with the fibrinogen‐derived peptide, KQAGDV, had no effect on platelet adhesion, indicating that MMP‐13 binds αIIbβ3 closer to the primary RGD‐binding site. Soluble fibrinogen also did not block platelet adhesion to MMP‐13, in line with the need for platelet activation for soluble fibrinogen binding to αIIbβ3 to occur, whereas immobilized fibrinogen is already competent to bind. The GPVI‐specific scFv, 1C3, does not target the collagen‐binding site at the apex of GPVI, unlike 10B12, and was unable to inhibit the adhesion of washed platelets to CRP (Figure 1B). 1C3 binding requires both GPVI Ig domains and is thought to reduce platelet activation by inhibiting receptor clustering; its epitope includes isoleucine 148,^21^, ^23^ located in strand E on the opposite face of D2 to the crystal structure dimerization interface located in strand G.^24^ An indifferent control, the anti‐HLA‐A2 scFv, 2D4, was inactive in these experiments. In subsequent experiments, only low platelet binding was observed to isolated CAT and HPX domains of MMP‐13 in comparison with the intact protein (Figure 1C), indicating that neither domain alone governs the interaction between the MMP and platelets, and supporting the possibility that two sites on MMP‐13 cooperate to bind αIIbβ3 and GPVI.

**Figure 2:**
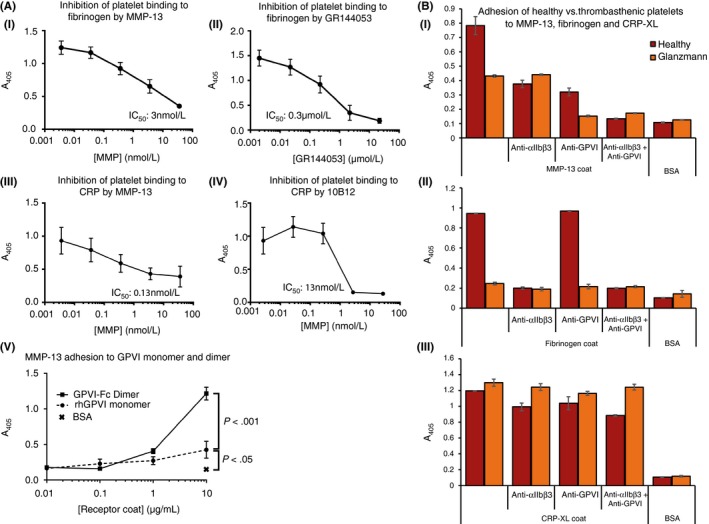
Competition, GPVI and Glanzmann platelet binding assays. (A) MMP‐13(E204A) and either 10B12 or GR144053 were used to obtain IC_50_ values for the inhibition of washed platelet adhesion to 10 μg/ml coated fibrinogen (i, iii) or CRP (ii, iv), respectively. (v) Adhesion of MMP‐13(E204A) to recombinant human GPVI monomer and dimer. Plates were coated with 10 μg/ml GPVI or BSA as a negative control. MMP‐13(E204A) at a concentration of 83 nM was allowed to adhere for 1 h at room temperature, then detected using an antibody directed at the MMP‐13 linker region, as described in Methods. Data represent mean A_450_ ± SE of three experiments. (B) Platelets from a healthy donor (red bars) and from a Glanzmann thombasthenic individual (orange bars) were allowed to adhere to MMP‐13, fibrinogen and CRP‐XL coated plates. Where appropriate, platelets were pre‐incubated with anti‐GPVI (1C3), or αIIbβ3 antagonists as described for Figure 1. Data represent mean A_405_ ± SE of duplicate readings for one experiment due to the rarity of the Glanzmann donor

Competition assays in which washed platelets were pre‐incubated with increasing amounts of the catalytically‐dead MMP‐13(E204A) provided further evidence that MMP‐13 interacts with both GPVI and αIIbβ3; like GR144053 and 10B12, MMP‐13 can compete αIIbβ3 off immobilized fibrinogen and GPVI off CRP (IC50 150 ng/mL and ~10 ng/mL respectively; Figures 2A[i‐iv]). Solid phase binding assays to coated isolated receptors revealed that MMP‐13 was able to bind weakly to GPVI monomer, but strongly to the GPVI dimer (Figure 2A[v]). Similar assays of adhesion to recombinant αIIbβ3 revealed some binding of its native ligand, fibrinogen, but little or no binding of MMP‐13, regardless of whether Mg^2+^, Mn^2+^, or Ca^2+^ was present, nor could we detect binding of MMP‐13 to purified αIIbβ3 (results not shown). Adhesion of αIIbβ3‐null Glanzmann platelets to MMP‐13, however, was markedly reduced (Figure 2B[i]). Blockade of αIIbβ3 on healthy platelets resulted in the same adhesion level as seen for αIIbβ3‐null platelets. As expected, binding of Glanzmann platelets to fibrinogen was abolished (Figure 2B[ii]) and to CRP was unaffected (Figure 2B[iii]). Our results indicate that, whilst MMP‐13 appears able to bind to αIIbβ3 on the platelet surface, recombinant αIIbβ3 used here cannot reproduce this effect.

**Figure 3:**
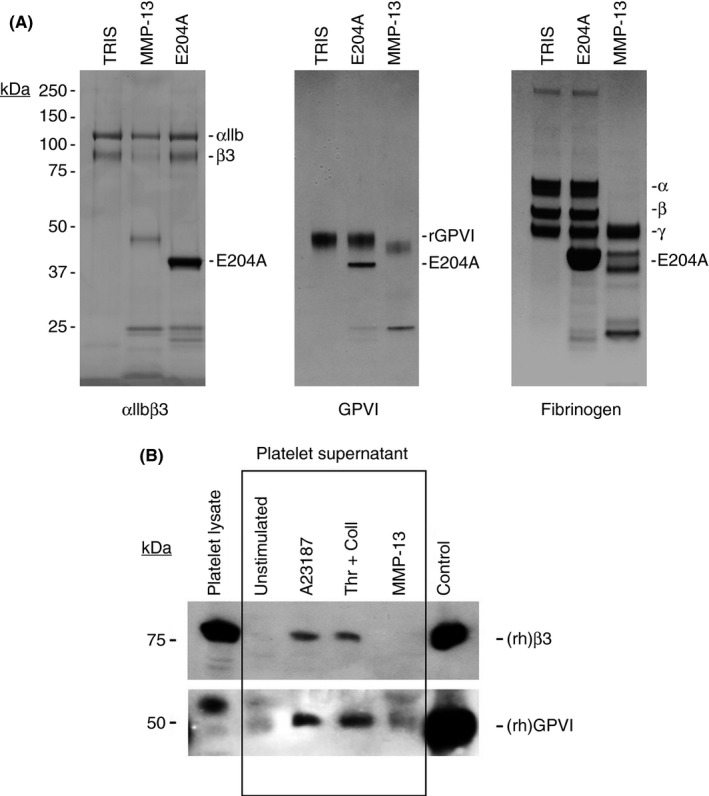
Platelet receptor cleavage and shedding assays. (A) Degradation analysis of recombinant platelet receptors by active and MMP‐13(E204A). Recombinant human (rh)GPVI and purified αIIbβ3 and fibrinogen type I were incubated with MMP‐13 or MMP‐13(E204A) for 2 h at 37°C. An equal volume of Tris buffer was used as a negative control. Samples were subjected to electrophoresis under reducing conditions and Coomassie stained. Images are representative of three experiments. (B) Shedding analysis of platelet receptors. Washed platelets were incubated with the calcium ionophore A23187, a thrombin and collagen type I mixture or MMP‐13 for 1 h at 37°C. The platelets were then pelleted, the supernatant isolated and subjected to SDS‐PAGE under reducing conditions and Western blotted. Platelet GPVI and the integrin β3 chain were detected using the appropriate antibodies as described in materials and methods. Recombinant human GPVI or αIIbβ3 were loaded onto the gels where appropriate as positive controls. Images are representative of three experiments

Whilst it was able to cleave the recombinant αIIbβ3 β‐chain and GPVI in solution, as well as fibrinogen α and β chains (Figure 3A), MMP‐13 was unable to either cause or mediate shedding of either receptor in situ (Figure 3B).

**Figure 4:**
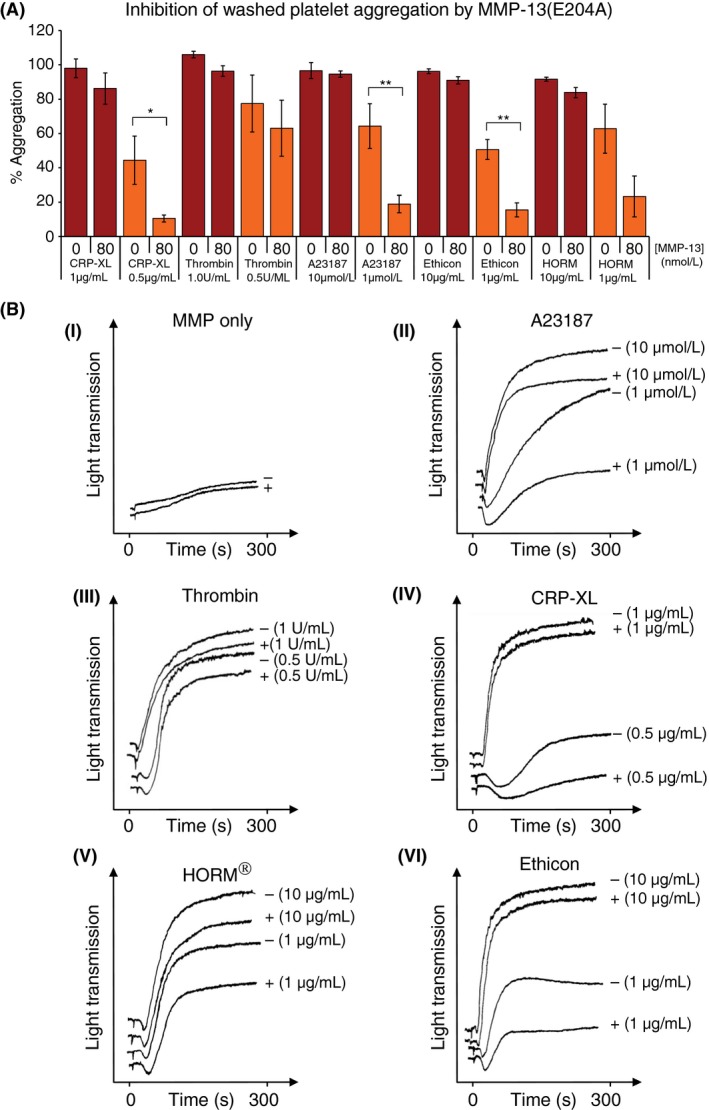
Inhibition of platelet aggregation by MMP‐13(E204A). Different agonists were added to washed platelets following pre‐incubation with 80 nmol L^−1^ MMP‐13(E204A). The equivalent volume of 0.01 mol L^−1^ acetic acid was used as a negative control. Mean donor responses performed in duplicate and repeated three times with different donors are shown in (A), and representative individual traces in response to MMP‐13 only, A23187, thrombin, cross‐linked collagen related peptide (CRP‐XL), HORM ^®^ and type I collagen shown in (B)

**Figure 5:**
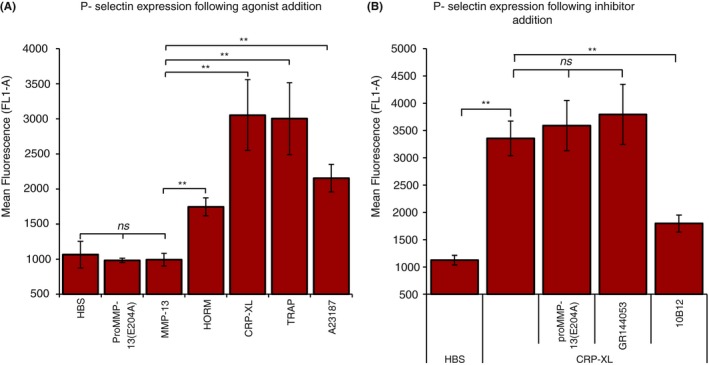
Activation of platelets in whole blood. Whole blood was mixed with anti P‐Selectin and the agonists 2 mmol L^−1^ proMMP‐13(E204A)/MMP‐13, 100 μg/ml CRP‐XL, 100 μg/ml HORM^®^ equine collagen I fibers, thrombin activating peptide (TRAP; 500 μmol L^−1^) calcium ionophore A23187 (100 μmol L^−1^) or HBS (negative control) added. Alexa 488 conjugated anti‐mouse was then added and after 10 minutes at 24°C the volume was made up to 500 μl with isotonic solution. After 30 minutes fluorescence was measured using an Accuri C6 flow cytometer (BD Biosciences, Oxford, UK). Data represent mean A_450_ ± SE of three separate donors. **P < .005; (one‐way anova and Holm multiple comparison test)

Pre‐incubation of washed platelets for 1 h with 80 nmol L^−1^ MMP‐13(E204A) significantly reduced platelet aggregation to a series of agonists, and for the mid‐range dose of each, analyzed using two‐way anova, the inhibitory effect of MMP‐13 was significant (*P *<* *.01). Prominent amongst these stimuli were: CRP‐XL, ionophore A23187, and bovine fibrillar collagen I, for which it was easier to establish mid‐range doses than for thrombin and equine fibrillar collagen. A summary of results is shown in Figure 4A and representative traces in Figure 4B. MMP‐13 does not activate platelets measured by flow cytometry: no change in fluorescence using the anti‐P‐Selectin antibody was observed following the incubation of whole blood with pro‐MMP‐13(E204A) or MMP‐13, whereas clear expression was seen following treatment with CRP‐XL, TRAP, HORM^®^, and ionophore A23187 (Figure 5A). In addition, MMP‐13 does not promote the aggregation of washed platelets (Figure 4B[i]). Subsequent flow cytometry experiments revealed that unlike the anti‐GVI scFv 10B12, neither proMMP‐13(E204A) nor GR144053 (a potent αIIbβ3 antagonist) are able to alter secretion following platelet activation via CRP‐XL (Figure 5B). This would suggest that in solution, the polymeric CRP‐XL is a more potent ligand than MMP‐13, and that the interaction of MMP‐13 with αIIbβ3 predominates over that with GPVI.

**Figure 6:**
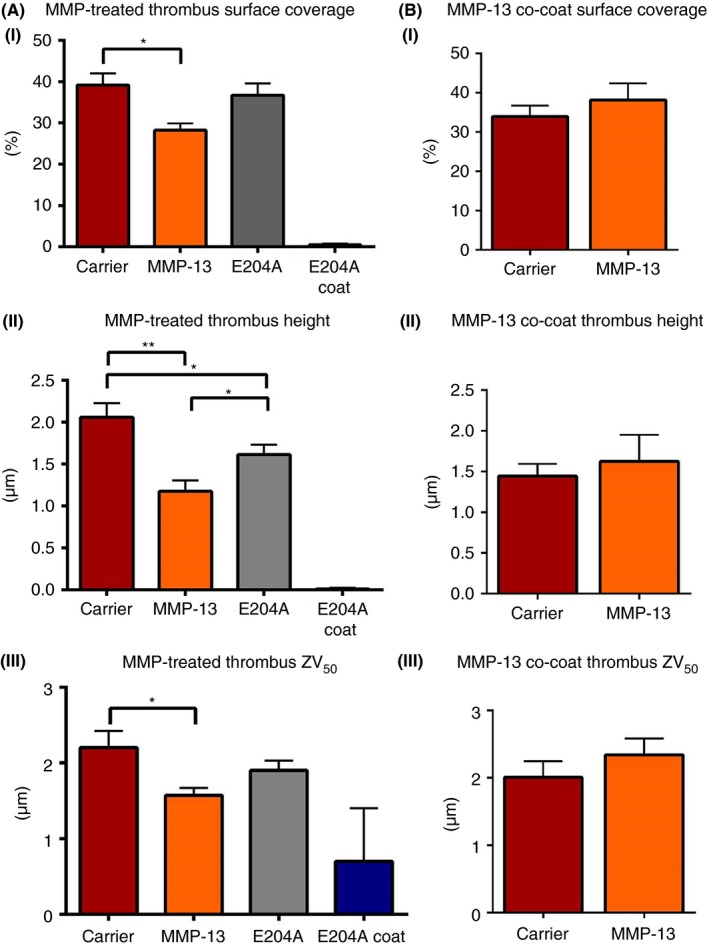
Platelet adhesion and thrombus deposition on fibrillar type I collagen. Untreated whole blood and blood pre‐incubated with 80 nmol L^−1^ MMP‐13 or negative control where stated was drawn through a flow chamber for 5 minutes over (A) collagen type I fibers or (B) collagen type I fibers co‐coated with MMP‐13 using a syringe pump to generate a wall shear rate of 1000 s^−1^, corresponding to arteriolar conditions. Surface coverage (i) mean height (ii) and (iii) ZV_50_ are the mean taken from a minimum of three different donors as measured using confocal microscopy. *P < .05; **P < .01; (one‐way anova and Holm multiple comparison test) relative to MMP‐untreated platelets

We investigated the influence of MMP‐13 or MMP‐13(E204A) on platelet adhesion and activation in flowing blood in vitro, using fibrillar collagen I coatings and a shear rate of 1000 s^−1.^ Pre–incubation of whole blood with MMP‐13 resulted in significantly reduced platelet surface coverage (*P *<* *.05), mean thrombus height (*P *<* *.01), and ZV_50_ (*P *<* *.05), using one‐way anova and Holm multiple comparison test; Figure 6A(i‐iii). ZV_50_ is the height within a Z‐stack at which thrombus volume = 50% and describes the activation state of adhered platelets in flowing human blood.^25^ Data obtained using pre‐incubations with MMP‐13(E204A) reached significance only for mean thrombus height (*P *<* *.05, Figure 6A[ii]). MMP‐13(E204A)‐coated slides were not able to support platelet adhesion under flow (Figure 6A[i‐iii]). These results indicate that the interaction of MMP‐13(E204A) with platelet GPVI and αIIbβ3 is sufficient to reduce platelet thrombus height. Catalytically active MMP‐13, whilst unable to cleave these receptors off the platelet surface, appears more able to inhibit platelet deposition. MMP‐13 co‐coated with collagen type I did not significantly alter platelet aggregate formation under flow conditions (Figure 6B[i‐iii]). Interaction of active MMP‐13 with other blood components is not excluded by the present work, and further study is indicated.

## 4 DISCUSSION

We have previously shown that degradation by MMP‐13 has the potential to modulate platelet adhesion to collagen.^17^ MMPs are zymogens; proteolysis is required to expose their catalytic site. Here we show that surprisingly, all forms of MMP‐13, pro‐ and active wild type enzyme as well as their catalytically inactive mutant counterparts, were able to support a high level of platelet adhesion under static conditions. This adhesion was inhibited by the anti‐GPVI scFvs 10B12 and 1C3 suggesting that the relatively large MMP‐13 occludes the sites of both 10B12 and 1C3 binding on the receptor. MMP‐13 was also able to bind strongly to the GPVI dimer. Although GPVI dimerization increases upon platelet activation, dimeric GPVI is also present on resting platelets and is required for their initial interaction with exposed collagen.^26^ Crystallography of the proMMP‐13 structure in complex with pro‐domain peptides revealed a dimeric form as an HPX‐mediated dimer like some other metalloproteinases, although in this study,^27^ MMP‐13 was not dimeric in solution. Conceivably, interaction of MMP‐13 with platelet surface GPVI dimer may provide a template for dimerization of the MMP. Platelet adhesion to MMP‐13 was also inhibited by the anti‐αIIbβ3 compound GR144053, and binding of Glanzmanns αIIbβ3‐null platelets to MMP‐13 was significantly reduced. Following pre‐incubation of washed platelets with MMP‐13, neither GPVI nor αIIbβ3 was shed from the platelet surface. It would appear, therefore, that whilst able to bind to platelet αIIbβ3 and GPVI, the orientation of MMP‐13 on the platelet surface does not allow access of its CAT domain to the cleavage site, which, for other sheddases, resides close to the transmembrane region and is regulated by membrane structure^28^ or substrate phosphorylation.^29^ Pre‐incubation with MMP‐13 did not result in platelet activation or aggregation. Here it is worth noting that MMP‐13 has been reported to cleave and thus activate PAR‐1 on cardiac cells.^30^ This has not been demonstrated on platelets, and may result in platelet activation concomitant with αIIbβ3 inhibition, however in this case the catalytically inactive proMMP‐13(E204A) is rendered unable to cleave the PAR‐1 receptor.

Coated as a substrate, MMP‐13 is independently unable to support platelet adhesion in whole flowing blood, and its colocalization with collagen does not result in an increase in platelet binding. In solution however, MMP‐13 is able to interact with platelet receptors GPVI and αIIbβ3 thereby modulating both platelet aggregation and thrombus formation under flow. Whilst MMP‐13 is able to compete with immobilized CRP‐XL for occupation of the GPVI receptor, our flow cytometry experiments reveal that the interaction of the MMP with platelets is insufficient to compete with the polymeric solution‐phase CRP‐XL and so alter platelet secretion. In this respect, it behaves much like the αIIbβ3 antagonist, GR144053, and it would appear therefore that the inhibitory effects of MMP‐13 in solution are mediated predominantly through αIIbβ3. At concentrations comparable to those reached in stroke patient plasma and found to correlate with severity of infarction,^31^ MMP‐13 can interact with both GPVI and αIIbβ3, and can compete with CRP and fibrinogen for occupation of these receptors. MMP‐13 is unable to cleave GPVI and αIIbβ3 from the platelet surface however, and appears to exert its effects by direct physical blockade of receptor engagement.

Until now, the role of MMP‐13 in atherothrombosis has been considered to be restricted to collagen proteolysis and remodelling, rendering plaque more friable and prone to rupture.^1^ However, MMPs are now emerging as important mediators of platelet function.^32^, ^33^ MMPs ‐1 and ‐2 are released from activated platelets where they colocalize with integrins at the sites of platelet–platelet interaction.^10^, ^34^ Active MMP‐1 and ‐2 can stimulate platelet function, suggesting receptor engagement and proteolysis.^34^, ^35^ MMPs in atherosclerotic lesions, released from the injured vessel wall itself or from platelets and monocytes, and that can also interact with platelets, are likely to interfere with the progression of plaque rupture, subsequent thrombosis and its associated pathologies including stroke, reperfusion injury, and hemorrhagic transformation. Indeed, these processes are associated with an upregulation of MMP activity.^2^, ^31^, ^36^ In mice, MMP‐13 is the key mediator of collagen degradation in atheroma and confers instability onto the vulnerability plaque cap.^37^, ^38^, ^39^ Disruption of the blood brain barrier (BBB) by MMPs is associated with hemorrhagic transformation following ischemic stroke,^36^, ^40^, ^41^ whilst MMPs ‐9 and ‐13 are implicated in the early pathology of stroke progression, and plasma MMP‐13 levels correlate with lesion volume.^2^, ^31^ In addition, the platelet collagen receptor GPVI has been identified in models of models of reperfusion injury,^42^ is associated with increased risk of stroke development, and is also seen after ischemic stroke.^43^

Here we demonstrate that MMP‐13 can exert an antithrombotic effect; inhibiting platelet aggregation and thrombus formation in flowing whole blood. It may be that this metalloproteinase has multiple roles in the pathology of ischemic stroke; firstly by undermining the stability of the fibrous cap of atheroma and so promoting its rupture, then modulating the BBB to increase bleeding risk, and finally acting on platelets to impair the aggregatory interactions, by antagonising GPVI and αIIbβ3 which would normally protect against bleeding. MMP‐13 would appear therefore to modulate the architecture around sites of infarction to increase both risk of stroke and its hemorrhagic complications. The effect of MMP‐13 will depend upon its local level and the exposure of MMP‐13‐binding matrix components and warrants further investigation.

## AUTHOR CONTRIBUTIONS

Vera Knäuper, Jean‐Daniel Malcor, and Stephanie Jung provided essential materials; Nicholas Pugh assisted with flow experiments; Richard Farndale designed the research; helped analyze the data and write the manuscript; and Joanna‐Marie Howes designed and performed the research, and wrote the manuscript.

## RELATIONSHIP DISCLOSURE

None of the authors have any disclosures relevant to this paper.
